# The Research on Operation of Obstructed Total Anomalous Pulmonary Venous Connection in Neonates

**DOI:** 10.1155/2014/576569

**Published:** 2014-06-26

**Authors:** Zheng Jinghao, Gao Botao, Xu Zhiwei, Liu Jinfeng

**Affiliations:** Department of Pediatric Cardiothoracic Surgery, Shanghai Children's Medical Center, Shanghai Medical College, Affiliated with Shanghai Jiaotong University, Shanghai 200127, China

## Abstract

*Objectives*. Total anomalous pulmonary venous connection (TAPVC) is a rare congenital heart disease. This study aimed to evaluate the outcomes of TAPVC repair in neonates, controlling for anatomic subtypes and surgical techniques. *Methods*. Between 1997 and 2013, 88 patients (median age: 16 days) underwent repair for supracardiac (31), cardiac (18), infracardiac (36), or mixed (3) TAPVC. All the patients underwent emergency operation due to obstructed drainage. Supracardiac and infracardiac TAPVC repair included a side-to-side anastomosis between the pulmonary venous confluence and left atrium. Coronary sinus unroofing was preferred for cardiac TAPVC repair. *Results*. The early mortality rate was 2.3% (2/88 patients). The echocardiogram showed no obstruction in the pulmonary vein anastomosis, and flow rate was 1.1–1.42 m/s in the 3-year follow-up period. *Conclusions*. The accurate preoperative diagnosis, improved protection of heart function, use of pulmonary vein tissue to anastomose and avoid damage of the pulmonary vein, and delayed sternum closure can reduce the risk of mortality. The preoperative severity of pulmonary vein obstruction, the timing of the emergency operation, and infracardiac or mixed-type TAPVC can affect prognosis. Using our surgical technique, the TAPVC mortality among our patients was gradually reduced with remarkable results. However, careful monitoring of the patient with pulmonary vein restenosis and the timing and method of reoperation should also be given importance.

## 1. Introduction

Total anomalous pulmonary venous connection (TAPVC) is a rare congenital heart disease, accounting for only approximately 2% of congenital heart diseases. The current remarkable results of TAPVC repair reflect the general improvements in preoperative diagnostic techniques, intraoperative techniques, and postoperative management strategies that have occurred over the last decade [1]. Symptoms of TAPVC occur early among infants, often causing deaths within 1 month after birth. Therefore, infants should undergo emergency surgical correction within the first month of life [2]. The aim of this study was to review and summarize the outcomes of emergency surgery for TAPVC and evaluate the postoperative risk factors.

## 2. Materials and Methods

Between March 1997 and May 2013, TAPVC was diagnosed in 463 infants who were admitted to our hospital, including 88 neonates with obstructive TAPVC. Among the patients, 49 were male and 39 were female, with ages ranging from 7 to 28 days (median: 16 ± 3.8 days). The weights varied from 2.9 to 7.0 kg (median: 4.2 ± 1.1 kg). Preoperative examination revealed a percutaneous oxygen saturation level of 71%–89% (median: 79% ± 20%). An echocardiographic examination was performed to evaluate the left ventricular systolic function in all of the cases. In addition, a computed tomography (CT) scan was performed in 12 cases. In 17 patients with cardiac dysfunction and serious internal environment disorder, including oliguria, lactic acid increased, metabolic acidosis, and hyperkalemia, cardiotonics and diuretics were administered to adjust the lactic acid level and surgery was performed 12–24 h after the lactic acid level dropped to 3–5 mg/mL. Diagnostic echocardiography was performed in the remaining infants; based on the diagnosis, emergency operation was performed within 24 h after admission to our hospital.

The 88 patients underwent repair for supracardiac (31), cardiac (18), infracardiac (36), or mixed (3) TAPVC. For the supracardiac TAPVC approach, dissection was performed in the dome of the atrium between the aorta and superior vena cava. Four pulmonary veins were detached, and side-to-side anastomosis was performed between the pulmonary venous (PV) confluence and the posterior left atrium. The posterior wall of the left atrium was opened parallel to the direction of the consolidated vein and opened upward to the roof of the left auricle and downward 3–5 mm from the mitral valve ring. The PV confluence was longitudinally opened to the initial position of the vertical vein, using 7-0 polypropylene running sutures to anastomose the posterior wall of the left atrium to the PV confluence. The anastomosis should extend to the branches of the left and right pulmonary veins if the PV confluence was narrow. To enlarge the left atrium, the atrial septal defect (ASD) or foramen ovale in most of the infants was closed with a pericardial patch. The vertical vein should be routinely ligated after the operation.

Among the infants with cardiac TAPVC at the cardiac level, 6 had pulmonary venous connection to the coronary sinus, 2 had normal connection to the right atrium, and all of the infants had patent foramen ovale. In the patients with coronary sinus connection, the dome of the coronary sinus was removed and the ostium sinus coronarii was connected with the ASD to form a large aperture between the left atrium and coronary sinus. The ASD was closed with a pericardial patch, and the ostium sinus coronarii was also sealed. In the patients whose pulmonary vein was directly connected to the right atrium, the foramen ovale was expanded to form an ASD, followed by drainage of the blood flow into the left atrium using a baffle.

Most of the pulmonary veins in the infracardiac TAPVC cases were drained from the posterior to left atrium into the PV confluence, separating the vertical vein downward to the mediastinum. We opened the posterior wall of the left atrium parallel to the direction of the vertical vein, opening it upward to the roof of the left atrium and downward 3–5 mm from the mitral valve ring. Longitudinally opened, the vertical vein was anastomosed to the posterior wall of the left atrium and vertical vein, using 7-0 polypropylene running sutures, and ligated at the level of the septum transversum.

In 3 mixed-type TAPVC cases, the pulmonary vein was drained into the coronary sinus, with the blood flow in remaining vein drained into the left innominate vein. The approach used in the 3 cases was similar to that used in the cardiac TAPVC cases, distally dissecting and turning downward the remaining PV or creating a new anastomosis to the left auricle.

The mean cardiopulmonary bypass (CPB) time was 88.9 ± 12.0 min (range: 65–156 min), and the mean aortic cross-clamp time was 48.9 ± 7.0 min (range: 21–116 min). Total circulatory arrest was used in 6 deep hypothermic circulatory arrest (DHCA) infants (duration: 31–44 min), with a CPB time of 46–72 min. Postoperative esophageal ultrasound examinations were performed in 56 infants, 2 of whom underwent a second bypass operation because of a PV flow velocity > 1.6 m/s, which decreased to <1.4 m/s after the anastomotic site was expanded.

## 3. Results

All of the operations were successful. However, 2 patients (2.9%) with infracardiac TAPVC died after the operation because of low cardiac output syndrome and bleeding, respectively. Six infants had delayed sternal closure because of postoperative cardiac intumescentia, with a mean closure time of 2–5 days. The mean mechanical ventilation time in the infants who survived was 6.0 ± 3.5 days (range: 2–13 days). Of those who survived, 7 had pulmonary hypertension crisis but improved after treatment with inhaled nitric oxide, Viagra, and bosentan under sedation, and 5 had low cardiac output syndrome and little or no renal output but improved after 2–5 days of peritoneal dialysis. The other complications included lung infection in 3 cases, suspected fungal infection in 1 case, pulmonary edema in 3 cases, pleural effusion in 2 cases, and pneumothorax in 2 cases. The mean duration of intensive care unit stay was 8.0 ± 2.9 days (range: 5–19 days).

The follow-up period ranged from 6 months to 3 years. The patients underwent ultrasound examination every 6 months after hospital discharge, and no residual shunt and obvious anastomotic obstruction were observed, with a PV blood flow velocity of 1.1–1.42 m/s. From the follow-up echocardiography, the right PV blood flow velocity was observed to increase to 2.2 m/s in 2 cases and it remained higher than the reference range within the 3-year follow-up period. The postoperative heart function was remarkably improved in 58 cases, with the heart shadow narrowed sharply and the pulmonary congestion resolved. The right PV blood flow velocity decreased to 0.9 ± 0.2 m/s after 3 years of follow-up.

## 4. Discussion

### 4.1. Preoperative Diagnosis and Preparation

Obtaining an early diagnosis of TAPVC is difficult because TAPVC can occur in combination with most congenital heart diseases and with varied symptoms [3]. Echocardiography can delineate the anatomical types and is the most commonly used diagnostic examination method for TAPVC because it is accurate and safe. When necessary, infants are subjected to CT examination instead of cardiac catheterization to confirm the TAPVC type. In our study, all of the patients underwent surgical operation after the TAPVC type was confirmed by echocardiography. The position of the pulmonary vein in 12 patients was determined by CT examination. Some infants with PV obstruction may encounter complications, including progressive anaerobic infection, systemic hypoperfusion or progressive hemodynamic failure, and progressive metabolic acidosis in most cases, with a fatality rate of 50% 3 months after birth. Therefore, these infants should be treated with surgery as early as possible [4]. In particular, the obstruction in infracardiac TAPVC usually occurs where the vertical vein passes through the diaphragm and sometimes requires drainage of the venous connection into the liver parenchyma and is associated with rapid deterioration. Therefore, surgery should be performed immediately once the diagnosis is made. Congestive heart failure due to obstruction often occurs during the neonatal period and is not an operation-related risk factor of mortality.

For maximal reduction in pulmonary vascular resistance and improvement of oxygen transport, 15 patients were preoperatively intubated for mechanical hyperventilation with 100% oxygen. Prostaglandin can maintain the arterial duct opening, which can be used as a protective channel of a right-to-left shunt. Timely correction of metabolic acidosis should be performed to improve sensitivity to catecholamine drugs. If these measures do not improve oxygenation and systemic circulation perfusion, emergency or subemergency operation should be considered. For patients with persistent severe preoperative metabolic disorder, preoperative treatment with ECMO was demonstrated to be effective in correcting and stabilizing viscera function, as well as improving outcomes of infants in critical conditions [4].

### 4.2. Operation and Technical Discussion

The choice of operative technique, with or without DHCA, is based on individual surgeon preference. However, the objective of all of the technical steps is to connect the pulmonary veins to the left atrium, eliminate all abnormal connections, and correct any other associated abnormalities, including ASD.

### 4.3. Supracardiac TAPVC

In supracardiac TAPVC cases, the left and right pulmonary artery (PA) branches should be intraoperatively separated to adequately reduce anastomosis tension [5], and careful ligation of the vertical vein should be performed to avoid damaging the proximal left phrenic nerve. To reduce the risk of residual obstruction of PV anastomoses because of pocket-like contraction of patching materials, we recommend making a wide anastomosis between the PV confluence and left atrium, from the superior approach between the superior vena cava and ascending aorta. To enlarge the left atrium, the ASD or foramen ovale was closed with a pericardial patch.

### 4.4. Cardiac TAPVC

In coronary sinus connection repair, a right atrial incision is made, and the roof of coronary sinus is removed, preventing it to get too close to the tricuspid annulus; the ostium sinus coronarii is then connected with the ASD [6] to form a large aperture between the left atrium and coronary sinus. The ASD was closed with a pericardial patch, and the ostium sinus coronarii were also sealed. For the 2 patients whose pulmonary vein was directly connected to the right atrium, the foramen ovale was expanded to form an ASD, followed by drainage of blood flow into the left atrium using a baffle.

### 4.5. Infracardiac TAPVC

Although infracardiac TAPVC and the other types of TAPVC differ in physiological and clinical manifestations, their surgical repair procedures are similar. However, in infants with infracardiac TAPVC, the PV connection through the hepatic vein to the inferior vena cava and then to the right atrium is frequently complicated by obstruction, which results in infants turning blue, having low cardiac output, and rapidly deteriorating owing to multiple organ failure; hence, these infants must undergo timely surgical treatment once diagnosis is established [6, 7]. In a previous case, the surgeon performed infracardiac TAPVC repair by lifting the heart on the right and exposing and connecting the left atrium with the pulmonary venous confluence. However, this technique is difficult to perform manually and often leads to a high myocardium temperature, which is unfavorable to the protection of myocardium. We divided the inferior and superior venae cavae and separated the interatrial groove; exposed the posterior left atrial wall, pulmonary venous confluence, and vertical vein; adequately separated the PV branches and vertical vein; cut off and sutured the end of the vertical vein; and expanded the left atrium capacity by opening the proximal part upward and creating a side-to-side anastomosis to the posterior left atrial wall. If the vertical vein is not long enough, the incision can be extended to the thick side of the PV confluence to ensure an adequate anastomotic ostium [8, 9]. Postoperative management of PA hypertension is very important and requires continuous monitoring by a pulmonary piezometer. In our patients, PA hypertension was treated with sedation, hyperventilation, positive inotropic agents, cardiac afterload-reducing drugs, and inhaled nitric oxide or Ventavis in varying dose combinations.

However, controversy exists regarding whether the vertical vein should be ligated. To alleviate PV anastomosis hypertension, some authors proposed to leave the vertical vein open, which may later spontaneously close [6], while others were suspicious of the possibility because of the potential risk of heart failure because of a left-to-right shunt. In our studies, we left the vertical vein open in 3 supracardiac TAPVC cases owing to postoperative PA hypertension and treated the other cases with ligation or incision and suture. We suggested that when the blood pressure declined and left atrial pressure increased above 15 mmHg according to the pressure monitor after the cessation of CPB, the vertical vein should be open.

### 4.6. Prognosis

A large number of studies indicated [9] that TAPVC mortality rates decreased sharply from 10–30% in the 1970s to 5–9% in the early 1980s up to the present. The incidence rate of postrepair PV stenosis is 6–10%, which usually occurred in infracardiac or mixed-type TAPVC cases. To reduce the risk of TAPVC mortality, performing preoperative ultrasound examinations, improving intraoperative myocardial protection, creating a side-to-side anastomosis between the pulmonary venous confluence and left atrium, avoiding PV branch distortion, and delaying sternal closure should be ensured. Preoperative severity of PV obstruction and emergency operation time may be related to patient outcomes and to infracardiac TAPVC. The reoperation rate is decreased to lower than 5% after improvement of the surgical technique. Although recent retrospective studies indicate that the TAPVC operative mortality rate decreased along with good late outcomes, we still propose careful monitoring of patients with PV restenosis and ensuring proper timing and appropriate choice of technique for reoperation.
